# Iron deficiency is a common disorder in general population and independently predicts all-cause mortality: results from the Gutenberg Health Study

**DOI:** 10.1007/s00392-020-01631-y

**Published:** 2020-03-25

**Authors:** Benedikt Schrage, Nicole Rübsamen, Andreas Schulz, Thomas Münzel, Norbert Pfeiffer, Philipp S. Wild, Manfred Beutel, Irene Schmidtmann, Rosemarie Lott, Stefan Blankenberg, Tanja Zeller, Karl J. Lackner, Mahir Karakas

**Affiliations:** 1 Department of General and Interventional Cardiology, University Heart and Vascular Center Hamburg, Hamburg, Germany; 2 DZHK (German Center for Cardiovascular Research), Partner site, Hamburg/Kiel/Lübeck, Germany; 3 Centre of Medicine II (Statistics), University Medical Center, Johannes Gutenberg-University Mainz, Mainz, Germany; 4 DZHK (German Center for Cardiovascular Research), Partner Site Rhine-Main, Mainz, Germany; 5 Center for Cardiology, University Medical Center, Johannes Gutenberg-University Mainz, Mainz, Germany; 6 Department for Opthalmology, University Medical Center, Johannes Gutenberg-University Mainz, Mainz, Germany; 7 Preventive Cardiology and Preventive Medicine, Center for Cardiology, University Medical Center, Johannes Gutenberg-University Mainz, Mainz, Germany; 8 Center for Thrombosis and Hemostasis, University Medical Center, Johannes Gutenberg-University Mainz, Mainz, Germany; 9 Department of Psychosomatic Medicine and Psychotherapy, University Medical Center, Johannes Gutenberg-University Mainz, Mainz, Germany; 10 Institute for Medical Biometry, Epidemiology and Informatics, University Medical Center Mainz, Mainz, Germany; 11 Institute for Clinical Chemistry and Laboratory Medicine, University Medical Center Mainz, Mainz, Germany

**Keywords:** Iron deficiency, General population, Risk factor

## Abstract

**Background:**

Iron deficiency is now accepted as an independent entity beyond anemia. Recently, a new functional definition of iron deficiency was proposed and proved strong efficacy in randomized cardiovascular clinical trials of intravenous iron supplementation. Here, we characterize the impact of iron deficiency on all-cause mortality in the non-anemic general population based on two distinct definitions.

**Methods:**

The Gutenberg Health Study is a population-based, prospective, single-center cohort study. The 5000 individuals between 35 and 74 years underwent baseline and a planned follow-up visit at year 5. Tested definitions of iron deficiency were (1) functional iron deficiency—ferritin levels below 100 µg/l, or ferritin levels between 100 and 299 µg/l and transferrin saturation below 20%, and (2) absolute iron deficiency—ferritin below 30 µg/l.

**Results:**

At baseline, a total of 54.5% of participants showed functional iron deficiency at a mean hemoglobin of 14.3 g/dl; while, the rate of absolute iron deficiency was 11.8%, at a mean hemoglobin level of 13.4 g/dl. At year 5, proportion of newly diagnosed subjects was 18.5% and 4.8%, respectively. Rate of all-cause mortality was 7.2% (*n* = 361); while, median follow-up was 10.1 years. After adjustment for hemoglobin and major cardiovascular risk factors, the hazard ratio with 95% confidence interval of the association of iron deficiency with mortality was 1.3 (1.0–1.6; *p* = 0.023) for the functional definition, and 1.9 (1.3–2.8; *p* = 0.002) for absolute iron deficiency.

**Conclusions:**

Iron deficiency is very common in the apparently healthy general population and independently associated with all-cause mortality in the mid to long term.

**Graphic abstract:**

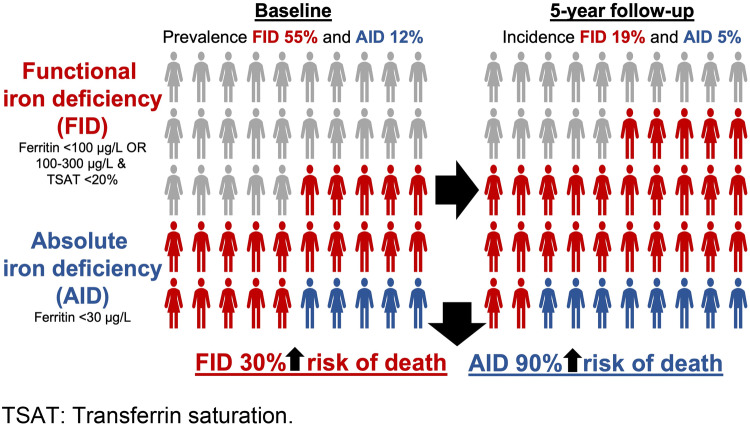

## Introduction

Iron is an essential trace element present in a number of molecular systems and plays a central role in oxygen transport and utilization as well as for mitochondrial function [1]. In clinical terms, prolonged iron deficiency often leads to anemia and subsequent symptoms such as dizziness and fatigue. In humans, intracellular iron is stored as ferritin, and a cut-off value of 30 µg/l is usually used for the diagnosis of an absolute iron deficiency [2]. However, ferritin is an acute phase reactant and its levels increase during inflammatory processes [3]. Transferrin is a negative acute phase reactant, and its levels decrease during inflammatory processes [4]. Transferrin saturation reflects the utilized amount of iron—i.e., the relative amount of transferrin that is loaded with iron [4].

Recently, iron deficiency has been recognized as an independent entity beyond anemia, with prognostic implications in diseased cohorts, mainly cardiovascular ones [5]. Moreover, a new functional definition of iron deficiency, combining low ferritin levels with a reduced transferrin saturation, was proposed and proved strong efficacy in randomized clinical outcome trials of intravenous iron supplementation and prospective studies [6–9]. Importantly, these trials and studies proved that the prognostic relevance of iron deficiency is irrespective of anemia [10].

However, there is a gap of knowledge on whether iron deficiency is a risk factor in the general population and which definition should be predominantly used for risk estimation.

The aim of this study is to characterize the impact of iron deficiency on all-cause mortality in the non-anemic general population based on two distinct definitions.

## Materials and methods

The study followed the recommendations of the Declaration of Helsinki and was approved by the ethics committee of the Chamber of Physicians of Rhineland-Palatinate, Germany (reference no. 837.020.07). Written informed consent was obtained from all participants.

### Study participants: Gutenberg Health Study

The Gutenberg Health Study (GHS) is a population-based, prospective, single-center cohort study initiated in 2007. Details on the study structure were published previously [11]. In short, study participants were randomly drawn from a governmental registry in the Rhine-Main region in the central western part of Germany. Individuals between 35 and 74 years were selected and invited to take part in a 5-h study visit at the University Medical Centre of the Johannes Gutenberg-University Mainz. Standardized interviews were performed to collect anthropometric data as well as data on lifestyle and cardiovascular risk factors. Furthermore, a standardized blood draw including material for bio-banking was taken. After 5 years, participants were again invited to a follow-up visit including all measurements described above. The primary endpoint was all-cause mortality verified from death certificates.

### Laboratory methods

Levels of ferritin, iron, and transferrin, as well as other laboratory parameters were measured routinely in blinded fashion, using automated immunoassays (Roche Cobas Integra 400, Basel, Switzerland). Anemia was defined as a hemoglobin level below 12 g/dl.

### Definitions of iron deficiency

For the present analysis, the following two definitions of iron deficiency were used:

1. Functional iron deficiency: ferritin levels ≤ 100 µg/l, or ferritin levels between 100 and 299 µg/l, if the transferrin saturation (TSAT) is below 20%. Hereby, TSAT is calculated using the following formula: (Iron {µg/dl}/transferrin {mg/dl}) *71.24. This is derived from randomized cardiovascular clinical trials such as the FAIR-HF trial [5] and the CONFIRM-HF trial [6], where intravenous iron supplementation documented therapeutic efficacy based on this definition.
2. Absolute iron deficiency: ferritin levels ≤ 30 µg/l. This definition is common within the internal medicine community [2].

### Statistical analysis

Continuous variables were described as mean (standard deviation) or median (1st quartile, 3rd quartile; if skewness > 1) and compared using the Wilcoxon signed rank rest. Categorical variables were described as absolute numbers and percentages and compared using the Fisher’s exact test. To evaluate the prevalence and cumulative incidence of iron deficiency within the general population, a cross table of iron deficiency at baseline and iron deficiency at follow-up was drawn. Fractional polynomial model was used to illustrate the association between age (continuous) and iron deficiency stratified by sex. Linear regression analyses for biomarkers as the dependent variable and iron deficiency as the predictor of interest were computed. To evaluate the impact of iron deficiency on all-cause mortality, Cox regression analyses were performed, adjusted for covariates that were either associated with iron deficiency or with all-cause mortality or with both (age, sex, hemoglobin, diabetes, hypertension, current smoking, obesity, dyslipidemia and family history of myocardial infarction/stroke) [12]. All analyses were run for both definitions of iron deficiency mentioned above and in men/women only. Linear regression analyses were used to assess associations of iron deficiency with blood parameters, adjusted for the same covariates as in the Cox regression model.

## Results

### Baseline characteristics of individuals with iron deficiency

Comparison of individuals with iron deficiency based on the two distinct definitions revealed some marked differences. As compared to individuals with absolute iron deficiency, individuals with functional iron deficiency were more likely male, were older and had a higher prevalence of cardiovascular risk factors such as hypertension or obesity. On the opposite, individuals suffering from absolute iron deficiency had lower hemoglobin values and were more likely to suffer from anemia. Table 1 displays detailed baseline characteristics on both groups.

**Table 1:** Baseline data of subjects with iron deficiency

	Functional iron deficiency (*n* = 2058)	Absolute iron deficiency (*n* = 495)	*p* value
Age (years)	54.7 (10.9)	48.5 (9.9)	< 0.001
Sex (Women)	63.0 (1296)	82.8 (410)	< 0.001
BMI (kg/m^2^)	25.9 (23.4, 29.3)	24.9 (22.1, 28.3)	< 0.001
Diabetes	9.3 (191)	7.3 (36)	0.19
Hypertension	47.0 (966)	33.1 (164)	< 0.001
Current smoking	20.7 (426)	17.4 (86)	0.10
Obesity	22.3 (458)	17.4 (86)	0.02
Dyslipidemia	41.8 (860)	26.5 (131)	< 0.001
Positive family history	23.2 (478)	24.2 (120)	0.64
Iron (µg/dl)	86.0 (68.0, 113.0)	84.0 (55.2, 112.0)	0.002
Ferritin (ng/ml)	75.53 (53.0, 99.0)	17.0 (11.3, 24.1)	< 0.001
Transferrin (g/l)	2.86 (0.4)	3.3 (0.5)	< 0.001
Transferrin saturation (%)	20.93 (16.94, 28.61)	18.36 (12.15, 25.36)	< 0.001
Hemoglobin (g/dl)	14.26 (1.08)	13.35 (1.28)	< 0.001
Anemia	1.1 (22)	10.5 (52)	< 0.001

### Prevalence of iron deficiency in the general population

**Fig. 1:**
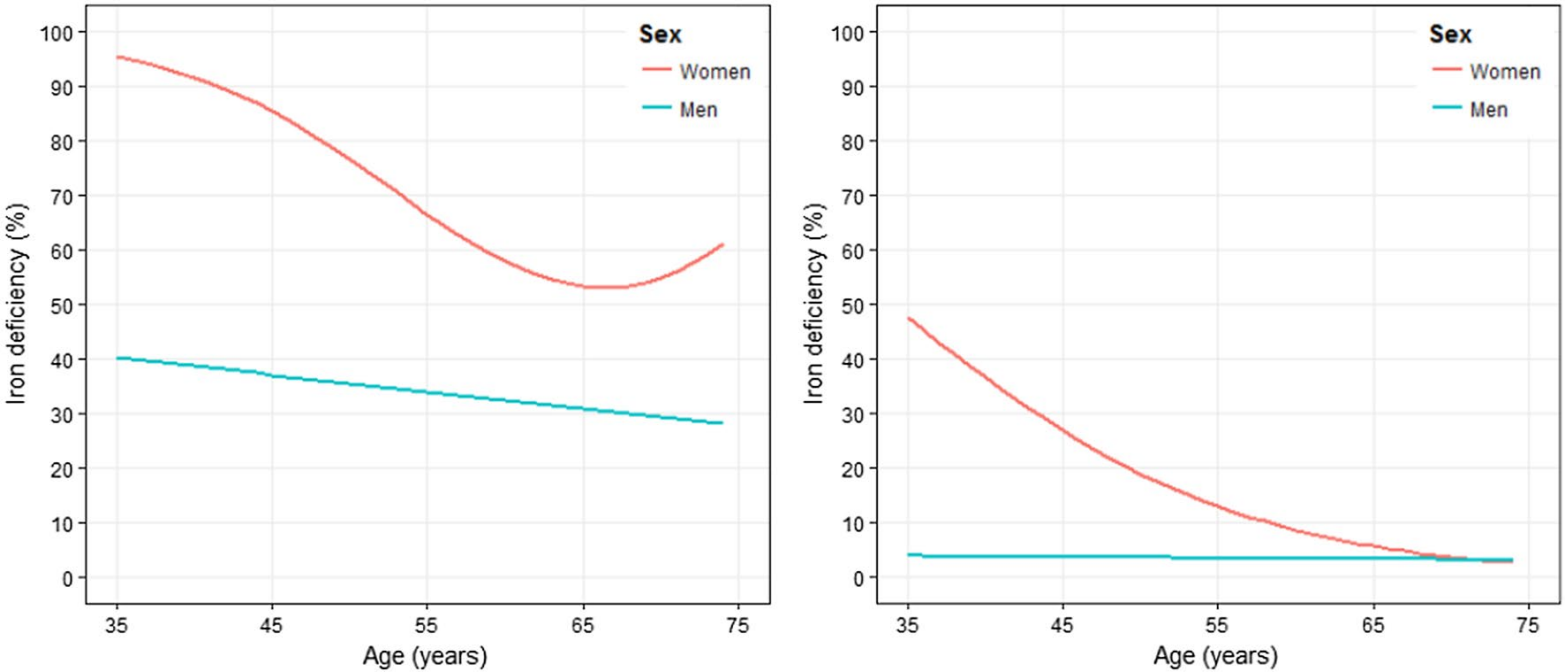
Functional (left) and absolute iron deficiency (right) and their association with sex and age

Among the 5000 participants of the GHS, overall prevalence of functional iron deficiency (estimates weighted for age and sex distribution of the study region) was 54.5 (53.2, 55.8) %, while absolute iron deficiency was present in 11.8 (10.8, 12.7) %. Stratification of the prevalence by age and sex reveals marked differences between men and women: With both definitions, the prevalence of iron deficiency is markedly increased in younger, menopausal, women. In older women, rate of absolute iron deficiency decreases, while functional iron deficiency increases beyond the age of 65 (Fig. 1). As expected, the prevalence of iron deficiency was increased in anemic subjects: functional iron deficiency: anemic women 89.7 (82.7, 96.8) %, non-anemic women 73.3 (71.7, 75.0) %, anemic men 76 (52.2, 99.8) %, non-anemic men 34.8 (32.8, 36.9) %; absolute iron deficiency: anemic women 71.3 (60.8, 81.8) %, non-anemic women 18.4 (16.8, 20.1) %, anemic men 25.6 (0.5, 50.6) %, and non-anemic men 3.3 (2.5, 4.0) %).

### Incidence of new diagnoses of iron deficiency

A total of 1951 individuals out of the 5000 had no functional iron deficiency at baseline. Of these individuals, 361 (18.5%) were newly diagnosed 5 years later during the planned follow-up visit. Regarding absolute iron deficiency, out of 3701 individuals without iron deficiency, 177 (4.8%) developed iron deficiency 5 years later.

### Association of iron deficiency with all-cause mortality

After a median follow-up of 10.1 (9.67, 10.5) years, rate of all-cause mortality was 7.2% (361 validated death cases). As shown in Table 2, both definitions of iron deficiency were significantly associated with all-cause mortality. This association was stronger with absolute iron deficiency. The magnitudes and directions of these associations were similar when stratified by sex (Table 2).

**Table 2:** Cox regression analyses for the association of iron deficiency and all-cause mortality

	Functional iron deficiency		Absolute iron deficiency	
	Hazard ratio	*p* value	Hazard ratio	*p* value
All individuals	1.29 (1.04, 1.62)	0.02	1.89 (1.26, 2.82)	< 0.01
Only men	1.30 (0.99, 1.70)	0.06	1.97 (1.10, 3.53)	0.02
Only women	1.34 (0.90, 2.00)	0.15	1.77 (1.00, 3.14)	< 0.01

Cox regression models were adjusted for age, sex, hemoglobin, diabetes, hypertension, current smoking, obesity, dyslipidemia and family history of myocardial infarction/stroke

### Association of iron deficiency with blood parameters

As shown in Table 3, iron deficiency, regardless of definition, was associated significantly and to a relevant level with level of erythrocytes (*R*^2^ of 0.64 for both definitions) and level of thrombocytes (*R*^2^ of 0.09 for both definitions).

**Table 3:** Association of iron deficiency with blood parameters

	Functional iron deficiency		Absolute iron deficiency	
	Estimate	*p* value	Estimate	*p* value
Thrombocytes (bil./l)	10.0 (6.5, 14.0)	< 0.001	7.1 (0.75, 13.0)	0.03
Erythrocytes (bil./l)	0.08 (0.07, 0.1)	< 0.01	0.14 (0.11, 0.16)	< 0.001

## Discussion

We report three major findings: (1) iron deficiency is very common in the apparently healthy, non-anemic general population. A total of 54.5% of participants showed functional iron deficiency at a mean hemoglobin of 14.3 g/dl, while the rate of absolute iron deficiency was 11.8%, at a mean hemoglobin level of 13.4 g/dl; (2) a relevant proportion of subjects develop new iron deficiency during mid-term follow-up. At year 5, proportion of newly diagnosed subjects with functional iron deficiency was 18.5%, while 4.8% of initially non-iron-deficient individuals had developed absolute iron deficiency; (3) iron deficiency is strongly and independently associated with all-cause mortality in the long term. Rate of all-cause mortality was 7.2% (*n* = 361) after a median follow-up of 10.1 years. After adjustment for hemoglobin and major cardiovascular risk factors, the hazard ratio with 95% confidence interval of the association of iron deficiency with mortality was 1.3 (1.0–1.6; *p* = 0.023) for the functional definition, and 1.9 (1.3–2.8; *p* = 0.002) for absolute iron deficiency.

Anemia is the striking symptom of iron deficiency [13]. Therefore, iron deficiency was reduced to this context for decades, and definition of iron deficiency was limited to very low ferritin levels (usually below 30 ng/l). However, recent research indicates that the impact of iron deficiency goes way beyond subsequent anemia [14]. Ferritin is an acute-phase reactant that increases even during subclinical inflammatory conditions, and the true burden of iron deficiency may have been underestimated. Most renowned is the FAIR-HF trial, which evaluated the effect of iron supplementation on a functional endpoint (self-reported Patient Global Assessment, NYHA functional class, and 6-min walk test) as compared to placebo in individuals with chronic heart failure and iron deficiency. Importantly, this trial did not only show a benefit of iron supplementation on the primary endpoint, but also showed that this association was independent of the baseline hemoglobin levels [5]. The definition of iron deficiency in this trial and other cardiovascular trials differed significantly from the low ferritin definition used in previous studies, with transferrin saturation being added as a diagnostic parameter. The use of this definition was based on the finding that most cardiovascular diseases are associated with a systemic inflammatory response which might elevate ferritin levels and therefore obscure the low ferritin definition of iron deficiency [15]. The present study is the first to transfer the new definition of iron deficiency and the emerging knowledge of beneficial effects of supplemental therapy in those deemed functional iron deficient to the general population. Functional iron deficiency seems more accurate in terms of sex-specific prediction, since absolute iron deficiency is much more frequent in menopausal women. Additionally, this study observed an increasing prevalence of functional iron deficiency in older women, whereas prevalence of absolute iron deficiency in women and both forms in men showed a decreasing prevalence with older age. Although it could be speculated that this relates to hormonal changes in the menopause, the exact mechanism is currently not known and should be further investigated. More surprisingly, the association with mortality was very robust and independent of hemoglobin (used as surrogate for anemia). Experimental and epidemiological evidence proves that, beyond hematopoiesis, iron is required for proper immune function and plays a central role in oxygen transport and utilization as well as for mitochondrial function [1]. Iron deficiency has been shown to compromise cell-mediated immunity, decreasing T-cell numbers and proliferative response and potentially reducing macrophage activity [16], which may reduce host capacity to control infection. Nevertheless, whether it has the capacity to challenge other established and emerging inflammatory and immune biomarkers in prediction of primary and secondary risk is subject to future studies.

Despite all efforts and meticulous design of the GHS, the presented study is subject to some limitations. First of all, although the above-mentioned analyses were adjusted for known confounders, there might be other confounders which are not known or were not measured at all. Secondly, the results can only be seen as descriptive due to the observational design and a causation cannot be derived from this. Thirdly, information on iron intake (e.g., nutritional habits) or iron loss (e.g., menstruation bleeding) were not available and could, therefore, not be factored into this analysis. Lastly, the findings were derived from a population based in the central western part of Germany. Therefore, the findings might not be transferred to a developing country or region.

## Conclusion

Iron deficiency is very common in the apparently healthy, non-anemic general population and strongly and independently associated with all-cause mortality in the mid to long term.
